# The Role of Social Defeat in Neurological differences in Psychotic Patients

**DOI:** 10.1192/j.eurpsy.2023.440

**Published:** 2023-07-19

**Authors:** A. Malaviya, P. A. Lalousis, S. J. Wood, A. Bertolino, S. B. Borgwardt, P. Brambilla, J. Kambeitz, R. Lencer, C. Pantelis, S. Ruhrmann, R. K. Salokangas, F. Schultze-Lutter, E. Meisenzahl, N. Koutsouleris, R. Upthegrove

**Affiliations:** ^1^School of Psychology, University of Birmingham, Birmingham, United Kingdom; ^2^Department of Basic Medical Sciences, University of Bari Aldo Moro, Bari, Italy; ^3^Department of Psychiatry, University of Basel, Basel, Switzerland; ^4^Department of Pathophysiology and Transplantation, University of Milan, Milan, Italy; ^5^Department of Psychiatry and Psychotherapy, Ludwig Maxmilians University, Munich; ^6^Department of Psychiatry, University of Münster, Münster, Germany; ^7^Melbourne Neuropsychiatry Centre, University of Melbourne, Melbourne, Australia; ^8^Department of Psychiatry and Psychotherapy, University of Cologne, Cologne, Germany; ^9^Department of Psychiatry, University of Turku, Turku, Finland; ^10^Department of Psychiatry and Psychotherapy, Heinrich-Heine University Düsseldorf, Düsseldorf; ^11^Department of Psychiatry and Psychotherapy, Ludwig Maxmilians University, Munich, Germany

## Abstract

**Introduction:**

The social defeat hypothesis (SDH) suggests that a chronic experience of social defeat increases the likelihood of the development of psychosis. The SDH indicates that a negative experience of exclusion leads to an increase in the baseline activity of the mesolimbic dopamine system (MDS), which in turn leads to the onset of psychosis. Social defeat models have previously been produced using animal models and preclinical literature; however, these theories have not fully been tested in human clinical samples. There have been studies implying changes in brain structure due to social defeat interactions; however, research evidence is varied.

**Objectives:**

This study aims to uncover whether exposure to SoDe has an impact on brain structure. Furthermore, we hope to understand if these changes are relevant to other mental health disorders.

**Methods:**

698 (506 no SoDe, 191 SoDe) participants between the ages of 15-41 were recruited from the PRONIA-FP7 study. SoDe was measured from the self-reported questionnaires’ Bullying Scale’ and ‘The Everyday Discrimination Scale’. T1-weighted structural MRI data were processed; five 2 sample t-test analyses were carried out to compare the GMV differences in the entire sample and between the four groups.

**Results:**

The VBM analysis showed significant group interactions in the right thalamus proper when comparing participants who had experience SoDe to participants who had not experienced SoDe including all 4 groups along with left cerebral white matter differences. In the ROP subgroup, significant group interactions in the left cerebellum white matter were found along with right cerebral white matter, left cerebral white matter and right Thalamus proper.

**Conclusions:**

The findings suggest that there are significant group interactions in thalamus and cerebral white matter. This is in keeping with some previous research suggesting volumetric changes in the thalamus due to stress and psychosis. Similarly for white matter there is some evidence suggesting differences due to SoDe and psychosis. However, there is a scarcity of research in this area with different research suggesting distinctive findings and therefore the evidence is inconclusive. In the ROP group analysis significant group interactions were present in the cerebellum due to SoDe experience. There is research suggesting the cerebellum’s role in multiple different aspects like social interaction, higher-order cognition, working memory, cognitive flexibility, and psychotic symptoms, with every research suggesting multiple different things the role of the cerebellum in SoDe in the ROP population is in question. Nonetheless this large-scale research presents some interesting novel finding and leads the way to a new area of research. Further analysis will explore the relationship between groups on markers of stress (CRP) and neuroinflammation as potential mediation of the environmental effects of SoDe.

**Disclosure of Interest:**

None Declared

